# Age‐Related Increase in Anaphylaxis Severity Is Associated With Enhanced Sensitivity to Allergic Mediators

**DOI:** 10.1111/all.70082

**Published:** 2025-09-29

**Authors:** Daniel Brigger, Melanie Steinmann, Nadine Roos, Pascal Guntern, Oliver M. Würgler, Lukas Michaja Balsiger, Alexander Eggel

**Affiliations:** ^1^ Department for BioMedical Research University of Bern Bern Switzerland; ^2^ Department of Rheumatology and Immunology University Hospital Bern Bern Switzerland

To the Editor,

1

Anaphylactic shock is the most severe outcome of an immunoglobulin E (IgE)‐mediated allergy. This potentially life‐threatening reaction with multi‐organ involvement is characterized by a rapid hypersensitivity response to allergen exposure. Despite the well‐documented general decline in immune cell function associated with aging, referred to as immunosenescence [1], older adults often experience more severe allergic reactions as compared to younger patients [2, 3]. Given the persistently high incidence and burden of severe anaphylaxis [4] in conjunction with the rapidly growing elderly population [5], we sought to investigate the underlying mechanisms contributing to this age‐related increase in allergy severity.

First, we investigated whether the age‐related increase in anaphylaxis severity that has been observed in humans is reproducible in mice. For this purpose, we passively sensitized young and aged C57BL/6 wild type (WT) mice intravenously with 2,4,6‐trinitrophenyl (TNP)‐specific IgE (clone: MEB‐38), followed by intraperitoneal challenge with TNP‐ovalbumin conjugate (TNP‐OVA) on the next day (Figure 1A). Indeed, aged mice exhibited significantly stronger anaphylactic responses, evidenced by greater loss in body temperature (Figure 1B) and a larger area under the hypothermia curve (AUC) (Figure 1C), which is in agreement with earlier reports [6]. To our surprise, however, serum levels of allergic effector cell‐related mediators including IL‐4 and mMCP‐1 showed no significant difference between age groups (Figure 1D,E).

**FIGURE 1:**
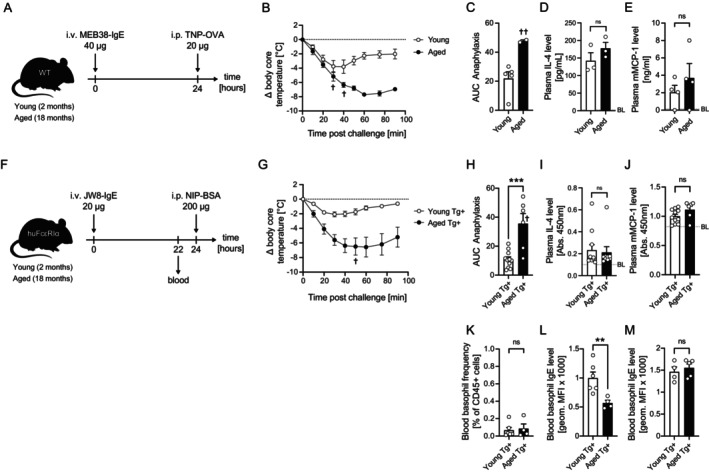
Both aged C57BL/6 wild‐type and human FcεRIα transgenic mice show more severe allergic reactions compared to young mice. (A) Passive systemic anaphylaxis (PSA) model in young and aged C57BL/6 WT mice. (B) Change in core body temperature (Δ*T*_0_, °C) was monitored for 90 min in young (*n* = 5) and aged (*n* = 4) mice. (C) Area under the curve (AUC) for temperature changes was calculated for each group. Plasma levels of IL‐4 (D) and mouse mast cell protease‐1 (mMCP‐1) (E) were measured by ELISA in young and aged mice (*n* = 3–4). Dashed gray lines indicate baseline levels (BL). (F) Passive systemic anaphylaxis (PSA) model using young and aged huFcεRIα mice. (G) Change in core body temperature (Δ*T*_0_, °C) was monitored for 90 min in young (*n* = 11) and aged (*n* = 7). (H) Area under the curve (AUC) of temperature changes was calculated for the respective groups. Plasma levels of IL‐4 (I) and mouse mast cell protease‐1 (mMCP‐1) (J) were measured by ELISA. Dashed gray lines indicate baseline levels (BL). (K) Blood basophil frequencies and surface‐bound IgE levels on basophils (L) were measured by flow cytometry in young and aged huFcεRIα mice. (M) Blood basophils were in vitro sensitized with 1 μg/mL JW8‐IgE, and receptor‐bound JW8‐IgE was quantified by flow cytometry. Data are shown as individual values with mean ± SEM. Young mice (2 months old) are represented in white; aged mice (18–19 months old) in black. **p < 0.01, ***p < 0.001; ns, not significant; † Marks case of fatal outcome per timepoint, †† Marks two cases of fatal outcome.

To further confirm these findings, we used humanized FcεRIα (huFcεRIα) mice sensitized with equal amounts of 4‐hydroxy‐3‐iodo‐5‐nitrophenylacetyl (NIP)‐specific human IgE (clone: JW8) followed by intraperitoneal challenge with NIP‐bovine serum albumin conjugate (NIP‐BSA) on the next day (Figure 1F). The pattern of exaggerated anaphylaxis in aged mice was consistent, with higher body core temperature loss and AUC values (Figure 1G,H). Despite generally increased body weight and blood volume in aged mice, no age‐related differences in serum levels of allergic effector cell mediators (i.e., IL‐4 and mMCP‐1) upon sub‐saturating antigen challenge were observed (Figure 1I,J).

To explore potential contributors to increased anaphylaxis severity in aged mice, we additionally quantified basophil cell numbers and the amount of NIP‐BSA‐specific IgE on blood basophils before antigen challenge. Interestingly, aged mice showed neither increased blood basophil counts nor higher IgE levels on these cells (Figure 1K,L). In contrast, the amount of NIP‐BSA‐specific IgE on blood basophils was significantly lower in aged compared to young mice (Figure 1L), while ex vivo sensitization with exogenous IgE exhibited comparable IgE loading on blood basophils from young and aged mice, indicating that the reduced in vivo IgE binding was due to sub‐saturating IgE concentrations (Figure 1M).

To further evaluate potential age‐related changes in allergic effector cell behavior, we passively sensitized young and aged huFcεRIα mice with JW8‐IgE and isolated peritoneal mast cells the next day to stimulate those with NIP‐BSA in vitro (Figure 2A). While we observed dose‐dependent activation as well as a decrease in degranulation and IgE cell surface level, no significant age‐related differences were apparent in these readouts (Figure 2B–G). To test whether basophils from aged mice might produce more IL‐4 upon activation, potentially enhancing vasodilation and exacerbating anaphylaxis, we generated bone marrow–derived basophils (BMBAs) from young and aged huFcεRIα mice. Cells were sensitized with JW8‐IgE and challenged with NIP‐BSA (Figure 2D,E) or ionomycin (Figure 2F) in the presence or absence of brefeldin A. No age‐related differences in activation or IL‐4 production were observed, consistent with serum IL‐4 levels measured after anaphylaxis. We further cultured bone marrow–derived mast cells (BMMCs) from young and aged WT and huFcεRIα mice to assess degranulation capacity in vitro. While proliferation and duplication times were comparable, aged BMMCs displayed reduced IgE receptor expression and diminished IgE‐dependent activation relative to young BMMCs (Figure S1A–E). Overall, these findings indicate that enhanced anaphylaxis severity in aged mice is unlikely to be driven by altered allergic effector cell function or increased IgE‐mediated degranulation capacity.

**FIGURE 2:**
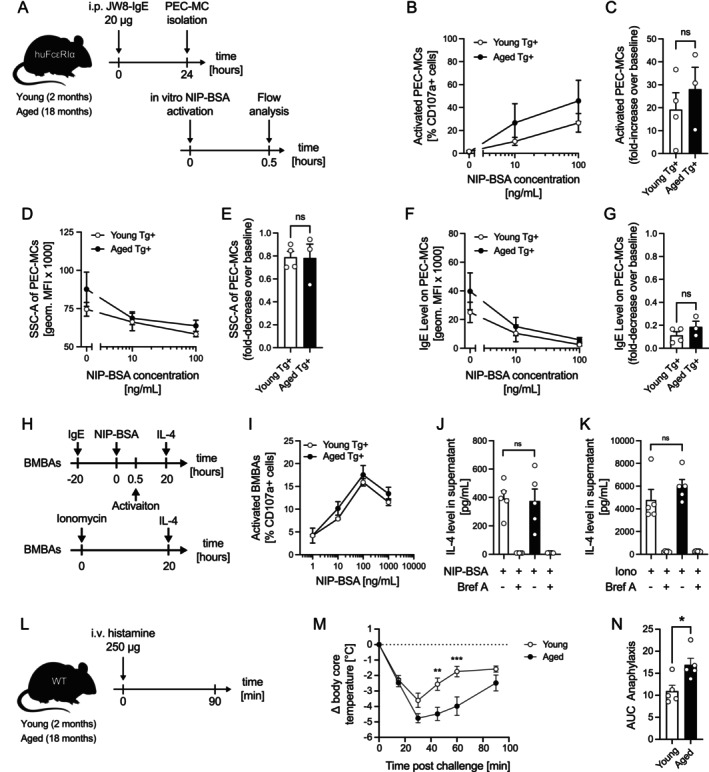
Enhanced allergic response in aged mice is due to heightened physiological responsiveness to mediators. (A) Young and aged huFcεRIα mice were sensitized according to the schematic. Isolated peritoneal mast cells (PEC‐MCs) were activated with increasing NIP‐BSA concentrations in vitro and degranulation has been assessed by flow cytometry by quantifying CD107a+ cells (B), degranulation by Side‐scatter (SSC‐A) (C) or decrease in IgE levels (D). Fold‐change of CD107a+ over baseline (C), degranulation (E) and IgE levels (G) of young and aged PEC‐MCs. (H) huFcεRIα BMBAs were sensitized overnight with 2 μg/mL JW8‐IgE and subsequently challenged with NIP–BSA conjugate. Alternatively, cells were stimulated with Ionomycin according to the schematic. (I) CD107a^+^ cells were quantified by flow cytometry. IL‐4 levels in culture supernatants following IgE‐dependent (J) or IgE‐independent (K) activation of BMBAs, assessed in the presence or absence of Brefeldin A (Bref A). (L) Schematic of in vivo mediator challenge. (M) Young and aged mice were intravenously injected with equal amounts of histamine, and core body temperature was monitored for 90 min (*n* = 5 per group). (N) AUC of temperature changes was calculated for both groups. Data are shown as individual values with mean ± SEM. Young mice (2 months old) are represented in white; aged mice (18–19 months old) in black. *p < 0.05, **p < 0.01, ***p < 0.001; ns, not significant.

Finally, to test the hypothesis whether aging might lead to an increased sensitivity to allergic mediators, we bypassed allergic effector cell activation and directly administered a fixed, sub‐saturating amount of exogenous histamine to young and aged WT mice (Figure 2G). Strikingly, aged mice showed significantly stronger anaphylactic responses (Figure 2H,I), indicating increased physiological sensitivity to allergic effector cell‐derived mediators. In summary, our findings reveal that age‐related increase in anaphylaxis severity is likely independent of altered effector cell activity or mediator release but is associated with an enhanced physiological responsiveness to allergic mediators such as histamine.

## Author Contributions

D.B., M.S., N.R., P.G., O.M.W., and L.M.B. conducted experiments and analyzed data. D.B. and A.E. conceptualized the study, interpreted data, and wrote the manuscript.

## Conflicts of Interest

A.E. is co‐founder, shareholder, and consultant of ATANIS Biotech AG and Excellergy Inc. All other authors declare no conflicts of interest.

## Supporting information


**Figure S1:** all70082‐sup‐0001‐FigureS1.pdf.

**Data S1:** all70082‐sup‐0002‐Supinfo1.docx.

## Data Availability

The data that support the findings of this study are available from the corresponding author upon reasonable request.
