# Supplementary figures and images for: Age‐Related Increase in Anaphylaxis Severity Is Associated With Enhanced Sensitivity to Allergic Mediators
Source: Allergy. 2025 Sep 29;81(3):910–3. doi: 10.1111/all.70082 (PMC12954550; doi:10.1111/all.70082)

Supplementary Figure 1

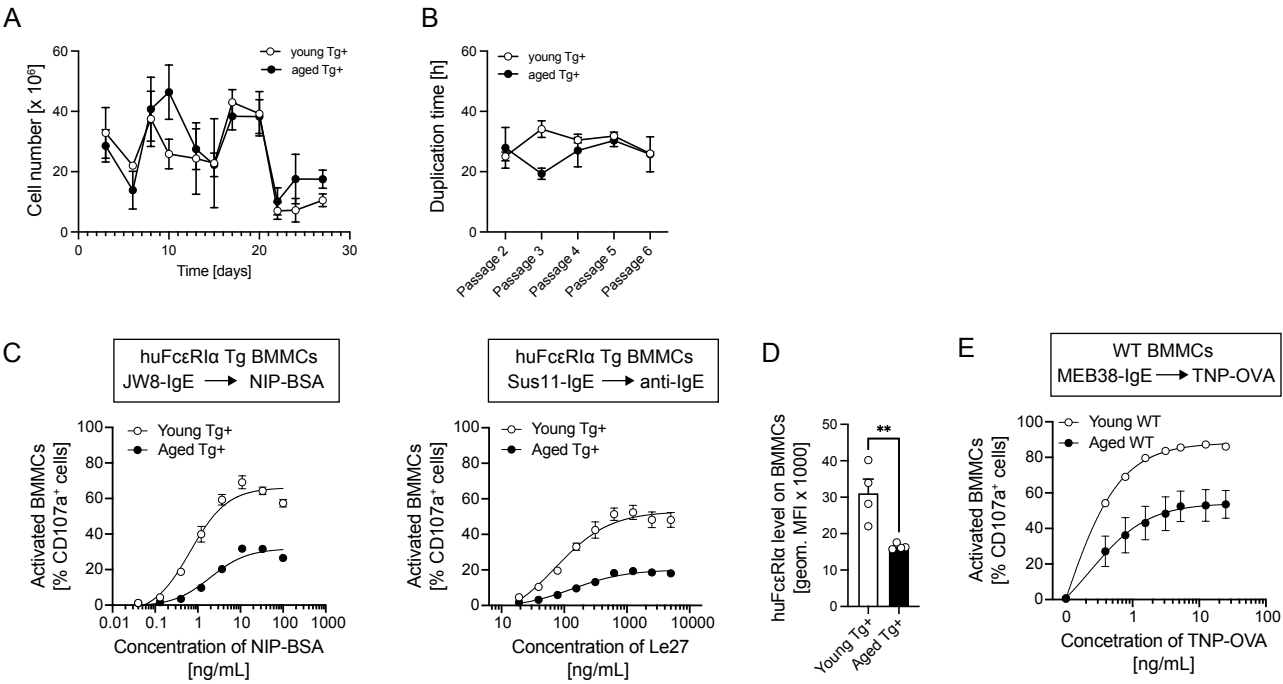

Supplement: Supplementary file 1 — Figure S1: all70082‐sup‐0001‐FigureS1.pdf. [file ALL-81-910-s002.pdf]
