# Supplementary material for: Age‐Related Increase in Anaphylaxis Severity Is Associated With Enhanced Sensitivity to Allergic Mediators
Source: Allergy. 2025 Sep 29;81(3):910–3. doi: 10.1111/all.70082 (PMC12954550; doi:10.1111/all.70082)
Supplement: Supplementary file 2 — Data S1: all70082‐sup‐0002‐Supinfo1.docx. [file ALL-81-910-s001.docx]

Supplementary Information

**Age-related increase in anaphylaxis severity is associated with enhanced sensitivity to allergic mediators**

**Material and Methods**

**Mouse strains and passive systemic anaphylaxis model.** Young (2 months) and aged (18 months) wild type (WT) C57BL/6JRj were purchased from Janvier Labs**.** Young (2-4 months) and aged (17-19 months) human FcεRIα (huFcεRIα) transgenic mice on a mixed C57BL/6J-C57BL/6N background were kindly provided by Prof. Jean-Pierre Kinet and bred in-house. Mice were housed in specific pathogen-free (SPF) conditions at the central animal facility of the Medical School of the University of Bern. Mice were passively sensitized by intravenous injection with 20 µg of NIP-specific IgE (clone: JW8-IgE; NBS-C BioScience, Vienna, Austria) or TNP-specific IgE (clone: MEB-38; Biolegend, San Diego CA). Blood was collected 20 hours post-sensitization by tail vein bleeding in plasma tubes (BD Biosciences, New York, NY, USA). Mice were challenged 24 hours post-sensitization with 200 µg NIP-BSA or TNP-OVA conjugate (LGC Biosearch Technologies, Hoddesdon, UK) by intraperitoneal injection. Body core temperature was repetitively measured for 90 minutes post-challenge using rectal thermometers (Vetronic Services LTD, Devon UK). Procedures involving animal subjects were performed under the Swiss ethical guidelines and approved by the local animal experimentation committee of the canton of Bern (BE42/22).

**Generation of bone-marrow derived mast cells.** Bone marrow-derived mast cells (BMMCs) were generated as previously described (1) . Briefly, mice were euthanized via CO₂ asphyxiation and bone marrow was harvested from one femur. Cells were cultured in BMMC medium consisting of RPMI-1640 with stable glutamine and 2.0 g/L NaHCO₃ (Seraglob), supplemented with 10% Hyclone FCS (Gibco), 100 U/mL penicillin, 100 μg/mL streptomycin (Gibco), 10 mM HEPES (1 M stock, Life Technologies), 1 mM sodium pyruvate (Gibco), 4 mM L-glutamine (Gibco), 1 x nonessential amino acids (Gibco), 30 ng/mL recombinant mouse IL-3 (PeproTech), and 50 μM 2-mercaptoethanol (Merck). During the first two weeks, the medium was changed every other day, and cells were maintained at a density of 2 x 10^6^ cells/mL in T75 flasks (Falcon) in a humidified 37°C incubator with 5% CO_2_. After two weeks, medium changes were performed twice weekly, and cultures were maintained at a density of 1 x 10^6^ cells/mL.

**In vitro activated BMMCs derived from young and aged donor mice.**

BMMCs were seeded to a density of 5 x 10^4^ cells per well of a 96 well U-bottom plate (Falcon), centrifuged for 5 minutes at 250 g and resuspended in 25 uL of fresh BMMC culture medium. They were incubated in a humidified 37°C incubator with 5% CO_2_ for 24 hours before being washed with phosphate buffer saline (PBS), centrifuged for 5 minutes at 600g and resuspended in 25 uL BMMC medium containing 150 ng/mL TNP-specific IgE (MEB-38, BioLegend) or 1ug/mL JW8 IgE (NBC118; NBS-Biosciences). The cells were re-incubated for 16 hours in a humidified 37°C incubator with 5% CO_2_. Subsequently, they were activated by addition of titrated TNP-OVA (LGC Biosearch Technologies, Hoddesdon, UK) and NIP-specific IgE (clone: JW8-IgE; NBS-C BioScience, Vienna, Austria), respectively, and simultaneously stained with 10 ug/mL of APC anti-CD107a staining antibody (1D4B, Biolegend) and re-incubated at 37°C for 25 minutes. The degranulation reaction was stopped by addition of ice-cold self-made FACS staining buffer (PBS, 2% FCS by Sigma, 0.05% Sodium azide by Sigma Aldrich) and the cells were centrifuged at 600g for 5 minutes. Cells were resuspended in FACS staining buffer and degranulation was measured by means of flow cytometric quantification of CD107a surface levels.

**Generation of bone-marrow derived Basophils (BMBAs).** Bone marrow-derived basophils (BMMCs) were generated as previously described (2) . Briefly, mice were euthanized via CO₂ asphyxiation and bone marrow was harvested from one femur. Cells were cultured in BMBA medium consisting of RPMI-1640 with stable glutamine and 2.0 g/L NaHCO₃ (Seraglob), supplemented with 10% Hyclone FCS (Gibco), 100 U/mL penicillin, 100 μg/mL streptomycin (Gibco), 10 mM HEPES (1 M stock, Life Technologies), 1 mM sodium pyruvate (Gibco), 4 mM L-glutamine (Gibco), 1 x nonessential amino acids (Gibco), 30 ng/mL recombinant mouse IL-3 (PeproTech), and 50 μM 2-mercaptoethanol (Merck). The medium was changed every 3 days, and cells were maintained at a density of 2 x 10^6^ cells/mL in T75 flasks (Falcon) in a humidified 37°C incubator with 5% CO_2_.

**In vitro activated BMBAs derived from young and aged donor mice.**

BMBAs were seeded to a density of 5 x 10^5^ cells per well of a 96 well U-bottom plate (Falcon), centrifuged for 5 minutes at 250 g and resuspended in 150 uL BMBA medium containing 2 µg/mL NIP-specific IgE (JW8; NBS-C BioScience, Vienna, Austria) IgE (MEB-38, BioLegend). The cells were re-incubated for 16 hours in a humidified 37°C incubator with 5% CO_2_. Subsequently, they were activated by addition of titrated NIP-BSA (LGC Biosearch Technologies, Hoddesdon, UK) and simultaneously stained with 10 ug/mL of APC anti-CD107a staining antibody (1D4B, Biolegend) and re-incubated at 37°C for 25 minutes. The degranulation reaction was stopped by addition of ice-cold self-made FACS staining buffer (PBS, 2% FCS by Sigma, 0.05% Sodium azide by Sigma Aldrich) and the cells were centrifuged at 600g for 5 minutes. Cells were resuspended in FACS staining buffer and degranulation was measured by means of flow cytometric quantification of CD107a surface levels. Alternatively, BMBAs were activated either in an IgE-dependent manner (NIP–BSA) or an IgE-independent manner (500 ng/mL ionomycin) for 20 hours, in the presence or absence of Brefeldin A (10 µg/mL). IL-4 levels in culture supernatants were quantified by ELISA.

**Cell preparation and flow cytometry.** Plasma was separated from blood collected in plasma tubes (BD Biosciences, New York, NY, USA) by centrifugation at 1000 g. Plasma was collected for further downstream assays. Cells were treated with ACK lysis buffer (150mM NH_4_Cl, 10mM KHCO_3_, 0.1mM EDTA Na_2_*2H_2_O dissolved in dH_2_O) following centrifugation at 600 g at 4°C. This step was repeated once and cell suspensions were stained with fluorochrome-conjugated antibodies for subsequent flow cytometric analyses. Peritoneal mast cells were isolated from the peritoneal cavity by lavage, stimulated in vitro with NIP-BSA, and analyzed by flow cytometry. Fc receptors were blocked with anti-CD16/32 (BioLegend) for 15 min at 4°C and cells were stained after a washing step with combinations of the following antibodies: anti-mouse CD19-biotin (1D3, BioLegend), anti-mouse CD4-FITC (L3T4, BDBioscience), anti-mouse CD3ε-biotin (145-2C11, BioLegend), anti-mouse TER119-biotin (TER-119, Biolegend), anti-mouse CD45-PerCP-Cy5.5 (30-F11, Biolegend), CD200R3-APC (Ba13 ,Biolegend), CD49b-PeCY/7 (DX5, Biolegend), anti-mouse IgE-FITC (RME-1, Biolegend), anti-human IgE-FITC (Ige21;ThermoFisher Scientifc) Streptavidin-BV650 (Biolegend). Blood basophils were acquired on a BD LSRII flow cytometer (BD Biosciences) and gated as singlets; lin^-^, CD45^inter^, CD200R3^+^, CD49b^+^ and IgE^+^ cells. Data were analyzed using FlowJo software (FlowJo LCC).

**Enzyme-linked immunosorbent assay (ELISA).** The chymotryptic protease mMCP-1 (Invitrogen by Thermo Fisher Scientific) and IL-4 ELISAs (Biolegend) were performed according to the manufacturer's instructions.

**Data and statistical analysis:** All data are expressed as mean ± standard error of the mean (S.E.M.), unless stated otherwise. All experiments were conducted in a randomized and blinded fashion. All treatment groups were compared for statistical analysis using GraphPad Prism 9.0 (GraphPad Software, Inc.). Figures were arranged and aligned using Adobe Illustrator (Adobe, Inc.). Two-tailed Student’s t-test was used to estimate statistically significant differences between two groups.  Statistical significance was calculated by one-way ANOVA followed by two-tailed post hoc Dunnett’s multiple comparison when 3 groups were involved. Wherever possible, data are shown as individual data points with mean ± S.E.M. * *P* < 0.05, ***P* < 0.01, ****P* < 0.001, *****P* < 0.0001.

**Supplementary Figure Legends**

**SFigure 1: Generation and evaluation of BMMCs from young and aged mice. (A)** Cell numbers of BMMC cultures from young and aged huFcεRIα mice over time. **(B)** Duplication time of BMMC cultures from young and aged huFcεRIα mice in different passages. **(C)** huFcεRIα BMMCs were sensitized overnight with 1 µg/mL JW8-IgE or Sus11-IgE and challenged with NIP-BSA conjugate or anti-IgE antibody (Le27); CD107a positive cells were quantified by flow cytometry. **(D)** Geometric mean fluorescence intensity (geom. MFI) of huFcεRIα protein was measured by flow cytometry on BMMCs **(E)** WT BMMCs from young and aged mice were sensitized overnight with 0.15 µg/mL TNP-specific (MEB-38) IgE and challenged with TNP-OVA conjugate. CD107a positive cells were quantified by flow cytometry.

**References:**

1. Zbären N, Brigger D, Bachmann D, Helbling A, Jörg L, Horn MP et al. A novel functional mast cell assay for the detection of allergies. *J Allergy Clin Immun* 2021;**149**:1018-1030.e11.

2. Sokol CL, Barton GM, Farr AG, Medzhitov R. A mechanism for the initiation of allergen-induced T helper type 2 responses. *Nat Immunol* 2008;**9**:310–318.
